# Physicians’ and Machine Learning Researchers’ Perspectives on Ethical Issues in the Early Development of Clinical Machine Learning Tools: Qualitative Interview Study

**DOI:** 10.2196/47449

**Published:** 2023-10-30

**Authors:** Jane Paik Kim, Katie Ryan, Max Kasun, Justin Hogg, Laura B Dunn, Laura Weiss Roberts

**Affiliations:** 1 Department of Psychiatry and Behavioral Sciences Stanford University School of Medicine Palo Alto, CA United States; 2 Department of Psychiatry University of Arkansas for Medical Sciences Arkansas, CA United States

**Keywords:** artificial intelligence, machine learning, ethical considerations, qualitative study, qualitative, ethic, ethics, ethical, perspective

## Abstract

**Background:**

Innovative tools leveraging artificial intelligence (AI) and machine learning (ML) are rapidly being developed for medicine, with new applications emerging in prediction, diagnosis, and treatment across a range of illnesses, patient populations, and clinical procedures. One barrier for successful innovation is the scarcity of research in the current literature seeking and analyzing the views of AI or ML researchers and physicians to support ethical guidance.

**Objective:**

This study aims to describe, using a qualitative approach, the landscape of ethical issues that AI or ML researchers and physicians with professional exposure to AI or ML tools observe or anticipate in the development and use of AI and ML in medicine.

**Methods:**

Semistructured interviews were used to facilitate in-depth, open-ended discussion, and a purposeful sampling technique was used to identify and recruit participants. We conducted 21 semistructured interviews with a purposeful sample of AI and ML researchers (n=10) and physicians (n=11). We asked interviewees about their views regarding ethical considerations related to the adoption of AI and ML in medicine. Interviews were transcribed and deidentified by members of our research team. Data analysis was guided by the principles of qualitative content analysis. This approach, in which transcribed data is broken down into descriptive units that are named and sorted based on their content, allows for the inductive emergence of codes directly from the data set.

**Results:**

Notably, both researchers and physicians articulated concerns regarding how AI and ML innovations are shaped in their early development (ie, the *problem formulation* stage). Considerations encompassed the assessment of research priorities and motivations, clarity and centeredness of clinical needs, professional and demographic diversity of research teams, and interdisciplinary knowledge generation and collaboration. Phase-1 ethical issues identified by interviewees were notably interdisciplinary in nature and invited questions regarding how to align priorities and values across disciplines and ensure clinical value throughout the development and implementation of medical AI and ML. Relatedly, interviewees suggested interdisciplinary solutions to these issues, for example, more resources to support knowledge generation and collaboration between developers and physicians, engagement with a broader range of stakeholders, and efforts to increase diversity in research broadly and within individual teams.

**Conclusions:**

These qualitative findings help elucidate several ethical challenges anticipated or encountered in AI and ML for health care. Our study is unique in that its use of open-ended questions allowed interviewees to explore their sentiments and perspectives without overreliance on implicit assumptions about what AI and ML currently are or are not. This analysis, however, does not include the perspectives of other relevant stakeholder groups, such as patients, ethicists, industry researchers or representatives, or other health care professionals beyond physicians. Additional qualitative and quantitative research is needed to reproduce and build on these findings.

## Introduction

### Background

Innovation in the field of machine learning (ML) within artificial intelligence (AI) is accelerating in medicine, with more US Food and Drug Administration (FDA) approvals for algorithms and devices in 2022 than in any prior year [1,2]. As algorithm research, tool development, and clinical implementation proceed, AI and ML innovations stand to benefit many domains of medicine, from enhanced classification systems for clinical diseases and syndromes to highly individualized patient care, encompassing prediction, diagnosis, and treatment [3,4]. In parallel, ethics governance has been recognized as a priority and standard for the advancement of AI and ML in medicine, with recent guidance emerging from working groups, expert meetings, and scholarly work [5-8]. There is now a wide agreement that a failure to anticipate ethical issues threatens to compromise public trust in medicine and, ultimately, its embrace of AI and ML and their promise to improve human health [9].

Attention to ethical challenges in medical AI and ML has increased sharply in recent years in response to evidence showing that clinical AI and ML tools may offer limited generalizability and reproducibility [10,11], low rates of successful clinical adoption [12], and algorithmic bias [13]. Although the accompanying risks may not always be immediately obvious, past examples teach us that premature clinical integration of innovative tools can lead to *runaway diffusion* of risks to patients in clinical research and routine care, ranging from reduced benefit of the tools to outright harms, which rapidly become harder to address as tools become more widespread and ingrained in clinical processes [8]. Critical and timely work in clinical ethics has emerged to proactively meet emerging challenges through the articulation of possible frameworks and recommendations and with the benefit of instructive early case studies [6,14-17]. McCradden et al [6], for example, proposed an oversight procedure for medical AI to help bridge the *AI chasm* created by the divergent ethical and methodological norms of the clinical and computer sciences.

In addition to this foundational work, there is agreement that predicting and meeting new ethical challenges will require seeking, analyzing, and incorporating the perspectives of professionals who work along the full pipeline of AI and ML innovation (ie, key stakeholders) [18-20]. Seeking the perspectives of stakeholders and generating knowledge based on their perspectives serves to test the veracity of the assumptions made about their views, identify human factors that could present barriers to implementation, and better understand clinical needs. Bringing awareness to the areas of translation where developers’ intentions may not align with the goals of end users may serve to minimize ethical “strain,” as noted by Char et al [21]. This work is critical for forming comprehensive ethical guidance and responding constructively to differing normative views on AI and ML innovation.

Early stakeholder research regarding medical AI and ML primarily sought physicians’ and patients’ views regarding AI and ML tool implementation in clinical practice. It has yielded insights into concerns such as generalizability, algorithmic fairness, and clinical fit, as well as a range of ethical concerns that remain unclarified or unaddressed [22-25]. For instance, clinicians have reported uncertainty about their ability to collaborate effectively with AI and ML tools in clinics, given the numerous time and resource constraints of clinical ecosystems [25]. Patients expressed reservations about consenting to share health data for AI and ML research purposes and resistance to prognostic AI and ML systems that determine treatment admission without provider-patient dialogue [22]. Other reported considerations include end-users’ perceptions of algorithms’ utility, the potential for overreliance on algorithms when performing clinical tasks, users’ lack of knowledge of the rules governing algorithms, and disruptions of existing clinical infrastructure, workflows, and configurations of care teams [15,16,23,26].

A major gap in the current stakeholder literature on ethics in AI and ML is that it has not frequently sought the perspectives of other key stakeholders such as AI and ML researchers and developers [27]. Furthermore, because most of this work has so far focused on perspectives regarding the implementation and use of specific clinical tools, few studies have analyzed stakeholder views on the ethical challenges that they perceive in other phases of the innovation pipeline, including conceptualization and development [28,29]. One noteworthy exception is an interview study of 19 informatics leaders at US academic medical centers, which found that leaders perceived efforts to build interdisciplinary consensus and define clinical needs as necessary before the clinical implementation phase [30]. Although input seeking from end users to inform upstream development has been conceived as potentially helpful in closing the implementation gap, stakeholder research has been underused as an empirical method for developing comprehensive ethics guidance [31].

### Objectives

Therefore, the purpose of this study was to describe the landscape of ethical issues that AI and ML researchers and physicians with professional exposure to AI or ML tools observe or anticipate in the development and use of AI or ML in medicine. This report is the first in a series of papers to describe findings from a larger study in which we conducted open-ended, in-depth interviews with multiple stakeholder groups, including AI and ML researchers, physicians, ethicists, and patients. In this study, we focus on the perspectives of AI and ML researchers and physicians with professional exposure to AI or ML. Through an open-ended discussion, we aimed to identify ethical considerations that may not be as frequently elevated in the literature, potentially because of a hyperfocus on already known issues. Given the lack of prior work involving these 2 stakeholder groups, we had no a priori hypotheses about common or divergent perspectives. Rather, we sought to describe the current landscape of ethical considerations.

## Methods

### Study Design

The purpose of this study, which is part of a broader project (National Center for Advancing Translational Sciences R01-TR-003505) studying the influence of AI and ML tools on clinical decision-making, was to describe the views of AI and ML researchers and physicians regarding ethical considerations they have encountered or anticipated in the development, refinement, and application of AI and ML in medicine [32]. A qualitative descriptive approach was applied in the design and completion of this study, as this method aims to describe specific experiences or perceptions using language directly from the data and is well suited for topics that have been minimally studied previously [33,34].

Semistructured interviews, which are a common method of data collection in qualitative research, were used in this study to facilitate in-depth, open-ended discussions [35]. The interview questions were intentionally broad in scope to allow participants the opportunity to address the topics that they personally found the most significant, as opposed to responding to topics defined a priori by our research team. As participants were not necessarily trained in ethics or familiar with the associated vocabulary, questions regarding the benefits, risks, and unintended consequences of AI and ML were included to encourage them to consider broader challenges related to their work that could potentially have ethical implications. Interview guides were tailored for each participant group (eg, physician and researcher), keeping in mind their professional backgrounds and contextual information. Ultimately, interview guides did not vary drastically from group to group (Textbox 1). After the first few interviews, the questions were slightly revised for clarity, based on feedback. Relevant follow-up questions were asked in response to participants’ replies to the primary questions.

**Open-ended questions asked in interviews.**

**Asked of researchers**

1. How would you describe your work? Can you give specific examples of recent work? What is the value of your work in the field?
2. What are some of your personal observations and experiences regarding the use of machine learning (ML) in medicine? Are there any special ethical issues you have encountered in the development of algorithms for medicine?
3. Do you have an example from your day-to-day work of algorithmic development that may have ethical implications?
4. Can you think of any unintended consequences in the application of ML algorithms in medicine?
5. Are there any other areas in the field of computer science that you work in that we have not covered yet in our conversation? Are there different ethical issues in this subfield compared with ML?
6. Do you believe that there are limits to what ML can accomplish in medicine?
7. What are your aspirations (or predictions) for your field? Do you anticipate any ethical issues?

**Asked of physicians**

1. How would you describe your work? Can you give me an example of what your average day looks like, or describe a few of the recent projects that you have been working on?
2. Can you describe any first-hand experiences that you have had using machine ML or artificial intelligence (AI) applications within health care?
3. What are your impressions or observations about the use of ML or AI applications in health care? Are there any special ethical issues that you have encountered or considered when it comes to using ML or AI applications in health care?
4. What do you think are some of the potential benefits of using ML or AI applications in health care? What do you think may be some of the unintended consequences?
5. What are your hopes or aspirations when it comes to ML or AI applications in health care? Do you anticipate any ethical issues?
6. How do you think the use of ML or AI applications will impact the jobs of doctors? Do you think it will have any impact on the patient-provider relationship?
7. What recommendations do you have for developers who are interested in creating ML or AI applications for the field of medicine?

### Participants and Procedures

A purposeful sampling technique was used to identify and recruit the participants. Purposeful sampling is common in qualitative description research and involves identifying and recruiting specific individuals who are especially knowledgeable about the topic being studied [36]. For this project, we sought to interview researchers who had experience in developing AI or ML tools for use in medicine, and physicians who had experience developing or using such tools. By consulting the relevant literature and seeking recommendations from experts in the fields of AI, ML, medicine, and AI ethics, we identified 61 candidates (33 researchers and 28 physicians) from 10 US academic institutions that met these criteria.

Recruitment e-mails containing details about our project and an electronic interest form were sent to these 61 potential participants. A total of 29 potential participants submitted an electronic interest form. Of these, 21 (10 researchers and 11 physicians) scheduled and completed an interview. Interviews continued until content saturation was reached, that is, when additional data did not lead to the emergence of new or original ideas or themes [37-39]. The final cohort of participants was affiliated with 6 different US academic institutions and represented a variety of academic departments, including medicine, biomedical informatics, engineering, computer science, radiology, psychiatry, and surgery. All participants in the researcher group held master’s degrees or higher in computer science or a related field, and all participants in the physician group held MDs. The complete demographic information of the participants is available in Table 1.

Web-based interviews were conducted between November 2020 and April 2021 using Zoom (Zoom Video Communications). A PDF copy of the institutional review board–approved informed consent form was sent to all potential participants before their scheduled interview date. On the day of the interview, the interviewers verbally reviewed the content of the informed consent form with potential participants and answered any questions before obtaining verbal consent and beginning the interview. Interviews were conducted by 1 of our team’s 4 trained interviewers and lasted 52 minutes, 6 seconds on average, ranging from 29 to 95 minutes (SD 15 min 54 s). The interviews were audio recorded. The participants were compensated in the form of an electronic gift card to appreciate their time and effort.

**Table 1:** Study population characteristics by the participant group^a^.

Characteristics			Researcher (n=10)		Physician (n=11)		Overall (N=21)
**Gender, n (%)**							
	Men	4 (40)		8 (73)		12 (57)	
	Women	6 (60)		3 (27)		9 (43)	
**Age (y)**							
	Value, mean (SD)	31.6 (3.91)		48.6 (17.7)		41.0 (15.7)	
	Value, median (IQR)	31.0 (27.0-37.0)		44.0 (30.0-93.0)		35.5 (27.0-93.0)	
**Race, n (%)**							
	African American or Black	1 (10)		1 (9)		2 (10)	
	Asian	4 (40)		5 (45)		9 (43)	
	White	4 (40)		3 (27)		7 (33)	
	Other	1 (10)		2 (18)		3 (14)	
**Ethnicity, n (%)**							
	Not Hispanic or Latino	10 (100)		9 (82)		19 (90)	
	Hispanic or Latino	0 (0)		2 (18)		2 (10)	
**Degree, n (%)**							
	Doctor of Medicine	0 (0)		5 (45)		5 (24)	
	Doctor of Medicine or Doctor of Philosophy	0 (0)		5 (45)		5 (24)	
	Doctor of Philosophy or equivalent	5 (50)		1 (9)		6 (29)	
	Master’s	5 (50)		0 (0)		5 (24)	

^a^Note: 1 participant did not report age. Ten physician participants were Doctors of Medicine; 1 was a Doctor of Philosophy clinical psychologist.

### Data Coding and Analysis

The interviews were transcribed and deidentified by the members of our research team. Data analysis was guided by the principles of qualitative content analysis [40]. This approach, in which transcribed data are broken down into descriptive units, which are named and sorted based on their content, allows for the inductive emergence of codes directly from the data set [41]. After the transcription of the interviews, open coding was performed for each transcript by 2 authors. The authors independently highlighted the substantive interview content and suggested descriptive codes for this content. The authors then met as a group to review and discuss these preliminary codes and refine their names and definitions. All transcripts were then rereviewed by the 2 authors and coded using preliminary codes. The authors compared the coded units, refined the code names and definitions, and drafted the final version of the codebook, which contained 30 descriptive codes derived directly from the content of the interviews.

The transcripts and codebook were uploaded to NVivo 1.0 (QSR International) for final coding, which was completed by a single author (KR) and reviewed by the principal investigator (JPK). The full team contributed to the analysis, which involved assessing the coded units and developing categories and themes that described the coded content.

### Ethics Approval

This study obtained approval from the Stanford University Institutional Review Board before the start of research (approved protocol # 58118).

## Results

### Overview

**Figure 1:**
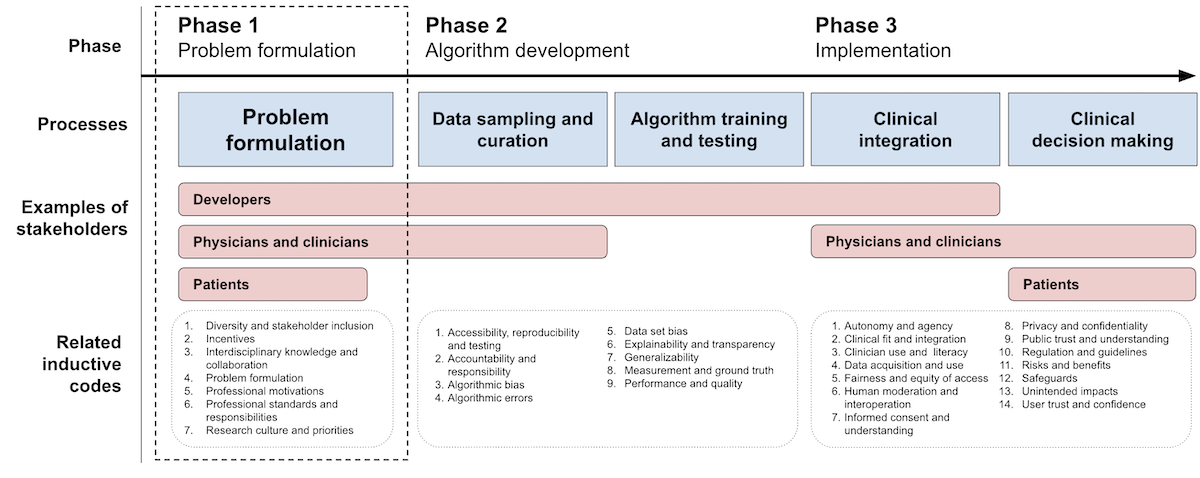
Phases of medical artificial intelligence and machine learning development as described by participants, and related inductive codes.

Qualitative content analysis was performed on the full data set, resulting in the identification of 30 inductive codes that described participants’ considerations relating to 3 distinct phases of AI and ML development for medicine: the problem formulation phase (phase 1), the algorithm development phase (phase 2), and the clinical implementation phase (phase 3; Figure 1).

Notably, 18 (86%) out of 21 researcher and physician interviewees addressed considerations related to phase 1. We describe this phase as the *problem formulation* phase, but it has been denoted in other literature as the *topic selection*, *need identification*, or *project definition* phase. This phase involves processes such as identifying health care needs that could be amenable to AI or ML solutions and formulating the scientific questions relevant to solving those needs.

**Figure 2:**
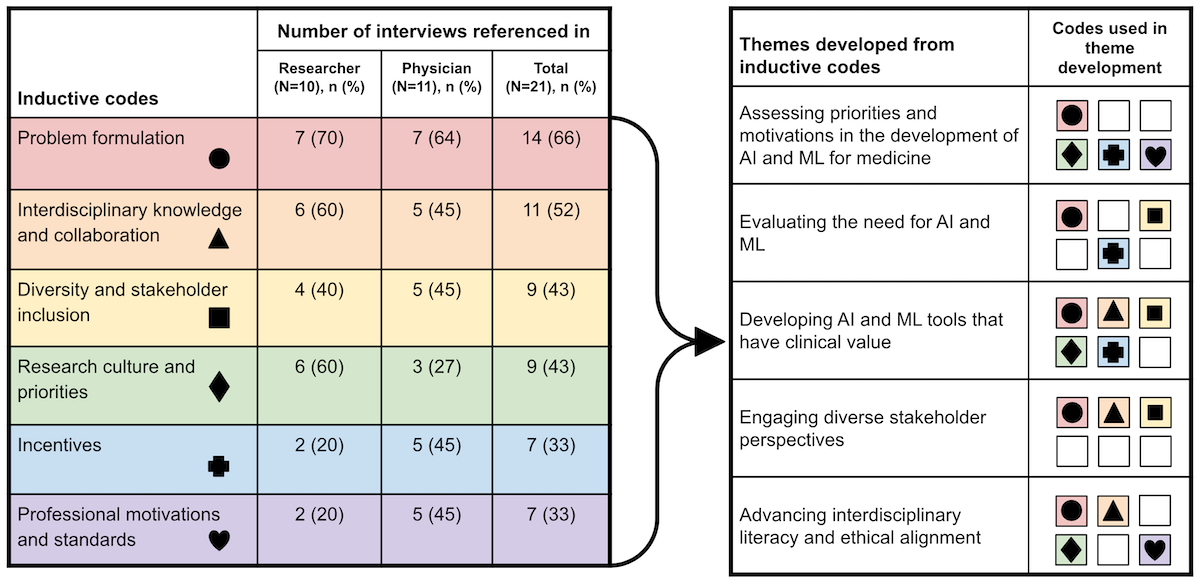
Inductive codes and themes describing researcher and physician views on phase 1 ethical considerations in medical artificial intelligence (AI) and machine learning (ML). For purposes of clarity, the codes “Professional motivations” and “Professional standards and responsibilities” were combined into a single row (“Professional motivations and standards”) for this figure.

Of the 30 inductive codes, 7 (23%) were primarily affiliated with phase 1; from these 7 codes, 5 major themes emerged (Figure 2). Within these themes, which are described in detail in this paper, interviewees identified a set of tightly interrelated phase-1 considerations that they perceived as having influence on the ethical dimensions of AI and ML research in medicine. Inductive codes and themes relating to phases 2 and 3 were also identified; due to the scope of the current paper, the analysis of these findings will be presented in a subsequent report.

### Assessing Priorities and Motivations in the Development of AI and ML for Medicine

When asked to discuss the ethical tensions they experienced or expected in medical AI or ML, both researchers and physicians described the extent to which different priorities between AI and ML and medicine could introduce ethical tensions as the 2 fields increasingly interact. As one researcher summarized, “the Silicon Valley ‘move fast, break things’ mantra doesn’t really work in healthcare” (participant 18, researcher). They drew clear contrasts between the priorities of AI and ML (ie, rapid innovation and development) and those of medicine (ie, reducing suffering and hardship associated with health disorders and conditions) and felt that value misalignment was especially likely to undermine innovation in the context of health care:

You’re dealing with a new technology, and you’re kind of straddling between innovation and patient care, and often those do not align with each other. You’re trying to, obviously, innovate to improve patient care, but at the same time, the innovation part of it may not necessarily be the best for the patient, or may not necessarily be the thing that is most needed at the moment. [Participant 15, physician]

Both physicians and researchers reflected on the nature of their research communities’ interests in AI and ML innovation, with some suggesting that they may sometimes be unduly influenced by factors unrelated to obligations to patient care. As one physician emphasized:

Everyone wants to think that their innovation is going to be the one that actually changes health care, but ultimately you have to be mindful of, “Am I doing this because I want to innovate or am I doing this because I really want to prove this one process or take care of the patient?” [Participant 15, physician]

Another researcher reflected on how the pursuit of innovation for its own sake can lead to AI and ML solutions that may yield technical and intellectual insights and a sense of accomplishment but may not always be grounded in or aligned with clinical needs:

The “so what?” also becomes part of the problem. I think a lot of machine learning people, myself included, have a tendency to think “Oh this is a great machine learning problem because...it is a really cool intellectual problem.” But I think it becomes a human problem once you think about the fact that this model could be deployed in the hospital. [Participant 10, researcher]

Several interviewees expressed wariness about the ease with which researchers and physicians may be motivated by their beliefs and attitudes regarding the promise and potential of AI and ML in medicine. One physician commented on how “often, with technologies like AI that garner a lot of attention and funding, there is a tendency to be driven by this desire to use the technology just because it’s a technology that’s interesting” (participant 15, physician). Another, looking back, saw their optimism decline somewhat over their research career:

Before I started doing any machine learning research I felt like machine learning was this sort of holy grail that was going to solve every research question. [Participant 22, physician]

### Evaluating the Need for AI and ML

Reflecting on the allure of AI and ML as an innovative field, interviewees expressed concern regarding the potential overproliferation of AI and ML tools in medicine for needs that could be better addressed with other technologies or interventions. Interviewees in both groups elevated the importance of performing an early assessment of the need for AI and ML and the value that it may add to specific medical contexts, that is, *“*Whether this is something that needs machine learning in the first place” (participant 22, physician)—as well considering, “At what cost is the question?*”* (participant 06, researcher) and whether a given AI or ML tool is “the right tool for the job” (participant 20, physician). One interviewee highlighted the importance of thinking about AI as one of many other innovative fields that can broadly apply to medicine in the interests of patients and humanity:

Think of AI as just an enabling technology like anything else. We don’t do anything for the sake of using the electronic health record. The electronic health record is a tool that allows us to take care of a patient, so AI should be the same thing. You should never do something for the sake of using AI. It should always be that we’re trying to solve this problem, we can’t solve it using existing tools so let's see if AI, if prediction, could allow us to formulate a better solution...You always have to ground yourself and then go back to, “this is just one of many technologies that I use, but ultimately I have to focus on solving the problem of taking care of the patient in front of me.” {Participant 15, physician}

### Developing AI and ML Tools That Have Clinical Value

Beyond initial assessments of the general need for AI and ML solutions, interviewees in both groups emphasized that it was just as important to evaluate the potential clinical value of the AI and ML tools under investigation. Several researchers expressed concerns about the proliferation of AI and ML tools that are not sufficiently evidence-based, that is, those created by developers who “[say] *that [they]’re going to save time in the clinic, and then that’s not possible—[They] have no evidence to show that that’s the case*” (participant 09, researcher). A recommendation to promote more robust AI and ML development proposed by members of both groups, in the words of a physician, was to “ground ourselves by being problem-focused” (participant 15, physician). In particular, they cautioned against presuming the benefits of AI and ML in a given problem in medicine, which they felt could reduce the likelihood of creating tools with clinical value:

We can’t just focus on building these new tools, but we need to think about the context in which they’re going to be used. We can’t think only about overall metrics...We really need to be prioritizing work that is actually meaningful, grounded in real problems as far as how machine learning has been used in healthcare. {Participant 33, researcher}

Interviewees in both groups referenced institutional and structural factors in research and academia that they felt could promote the development of tools that might not ultimately prove useful in clinical settings. Specifically, they discussed the impact of academic competition in the medical and AI and ML research communities, which is important for advancing a career but may not adequately prioritize the development of practical tools. Researchers and physicians agreed that “There is a disconnect because the traditional ways of academic advancement—publications, grants—reward publication of algorithms and capabilities, and papers. There are many, many, many algorithms, but we don’t see many translate into clinical practice” (participant 23, physician). They stressed their desire “to see the incentives in academia and elsewhere change so that people can really invest in solving real problems instead of just churning out these publishable units.” (participant 33, researcher).

Several researchers felt that the rapid advancement of AI and ML in medicine could paradoxically slow progress, with several mentioning that stepwise approaches to development should be prioritized (“Sometimes...we do not currently have the ability to arrive at a satisfactory solution...Sometimes we are aiming for [step] three or four and we should just start with one” [participant 13, researcher]). They posited that more fundamental AI and ML research may yield greater benefits in the field’s current state (“I tend to believe that the most “boring” work is the work that pays off the most” [participant 18, researcher]). Likewise, some observed that more fundamental work is often overlooked in favor of research topics which follow “the new trend” (participant 10, researcher). They expressed frustration that the AI and ML research community appears to prioritize and reward “trend-hopping” (participant 33, researcher), whereas the work needed to create benefit for clinical populations may be neglected or underfunded (“we’re probably not putting our energy in the right place” [participant 10, researcher]).

### Engaging Diverse Stakeholder Perspectives

Interviewees from both groups commented on the ways in which the composition of the research team—in terms of professional role (ie, developers, hospital administrators, physicians, and patients) and demographic characteristics (ie, race, gender, and ethnicity)—can impact the clinical utility of AI and ML solutions downstream:

Developers - definitely [work] with clinicians and communities and patients from the start. Because it's tempting, not just as a developer but also as a researcher, to feel like 'I have this really cool idea and I have this really cool algorithm and I'm just going to build it and then test it.' But that's a little bit of a disaster or a little bit of a risk of missing a lot of issues, or solving a problem that doesn't have to be solved, or not solving the right problem. {Participant 22, physician}

Interviewees in both groups emphasized the need to involve diverse collaborators “from the very beginning if we want to actually build something practically useful” (participant 13, researcher), with a special emphasis on including individuals who may have “certain lived experience, [who] are going to be able to identify some things that others wouldn’t see*”* (participant 14, researcher).

Multiple interviewees similarly addressed how the background and demographic composition of a research team can impact the types of AI and ML projects that are advanced in medicine, with one researcher commenting “I think a lot of it, for better or for worse, is motivated by personal excitement and motivation, and not direct thinking about what kind of problems we should be addressing. That reason to me is the most concrete motivation for why we should have diversity in the field” (participant 10, researcher). Other researchers emphasized the importance of considering “Who’s asking the questions?” (participant 14, researcher), as well as, including those “who haven’t had the privilege to ask the questions, who haven’t been empowered to be able to ask the questions” (participant 33, researcher). Some expressed concerns that a lack of diversity among investigators and research teams could skew research directions and minimize the concerns of underrepresented and marginalized groups:

We see a lot of that in machine learning: It’s not driven by what’s a real question in the communities...It’s driven by an idea that somebody had, an idea in a very homogenous team of people. {Participant 33, researcher}

### Advancing Interdisciplinary Knowledge and Ethical Alignment

Both developers and physicians commented on the need for greater collaboration between stakeholders in AI and ML and medicine, emphasizing that “there’s a big gap in those two communities in terms of the problems that one wants solved, the problems that are solved, how the communication happens, and how that’s all addressed” (participant 12, physicians). This physician noted that “deep collaborations are fairly rare...It’s not easy to find them” [12], in agreement with several researchers who described the current state of research as interdisciplinary on the surface, but still highly localized. Many interviewees perceived a need for greater interdisciplinary knowledge between the 2 fields, emphasizing the desire for “more people who are dual trained: who really deeply understand subject matter and who deeply understand algorithms” (participant 33, researcher), and who can “actually own a scientific question and try to answer it end-to-end” (participant 26, researcher). They felt that a greater commitment to interdisciplinary training and collaboration would lead to the development of more tools with clinical relevance and utility.

Interviewees highlighted how different approaches to ethical and professional standards in AI and ML research and medicine may be sources of conflict when applying AI and ML in medicine. They tended to agree that although research and patient care are united by institutionalized ethical commitments (eg, the Hippocratic Oath, the Belmont Report, and the Common Rule) and organizational safeguards (eg, institutional review boards), ethics in the emerging fields of AI and ML lack institutionalized guidance and typically consist of individual researchers voluntarily following informal guidelines and recommendations. Several researchers expressed the feeling that “Right now the system relies on people like me doing the right thing.” (participant 10, researcher) and felt that ethics are not adequately prioritized in computer science training and curricula:

We are taught to think, “Here is a thing that your program should do, and then if it does it then you’re good.” But you're not really taught to think about, what are all the other things that it could be doing as well on the side? We’re just biased towards getting the one positive result out of our program without thinking about all the negative consequences that could happen. {Participant 06, researcher}

Interviewees identified more training in ethics as necessary to support the translation of AI and ML research into robust clinical tools. Several researchers related a desire for enhanced ethical training among developers; one asserted the importance of “incorporating ethical thinking into every single class that computer scientists take, so that it is not just the one throwaway class you have to sit through, but it is like every time you do something, just think about [ethics] as well. Because the whole point is you should...think about the ethics and think about the potential backfiring while you’re designing the technology—not after you've designed it” (participant 06, researcher).

Interviewees also perceived a need for increased computer science education in medical training, noting that “there has to be the kind of literacy about computer science that is not currently required in the medical curriculum” (participant 10, researcher). Several physicians expressed similar desires, with one asserting, “We’re going to have to learn something about how these algorithms work...We’re going to own AI just as we've kind of owned other kinds of new technologies that have been incorporated into our practice” (participant 14, physician).

## Discussion

### Background

In this report, we sought the perspectives of researchers and physicians regarding ethical considerations in the translation of ML technologies into medicine. Existing qualitative literature pertaining to medical AI and ML has primarily focused on clinicians’ views on specific uses or implementations of AI and ML in medicine [12,16,22-24,26]. Our study is unique in that its use of open-ended questions allowed interviewees to explore their sentiments and perspectives without overreliance on implicit assumptions about what AI and ML currently are *like*. Because of the open-endedness of the questions, participants articulated the issues that they resonated with most strongly, as opposed to responding to prescriptive questions about issues defined a priori by our research team. To the best of our knowledge, this study is among the first to describe such perspectives.

Our findings revealed a range of ethical concerns shared by both researchers and physicians regarding the initial phase of research, which we have referred to as the “problem formulation” phase or “phase 1” (Figure 1). Although our interview questions did not specifically probe these early issues, most interviewees discussed them in great detail. Their concerns revolved around several broad themes (ie, influences on research directions, clinical needs and utility, stakeholder involvement, and interdisciplinary knowledge); interviewees viewed themes as interlinked and deserving of critical consideration before the beginning of algorithm development.

### Establishing Clinical Need and Value: The Significance of Phase-1 Decision-Making

To date, a small percentage of AI and ML tools developed for use in medicine have been successfully implemented in clinical practice, and for those tools that have been implemented, their acceptability has sometimes been disputed by health care practitioners and administrators [6,7]. For example, clinicians have raised concerns regarding the risks of cognitive burden, overreliance on algorithms, degradation of human clinical abilities, and patient overtreatment in response to several early sepsis detection tools [14-16,42]. Interviewees in our study were aware of this “AI chasm,” and identified processes that take place during phase 1, such as selecting a research question and building a research team, that they felt contribute to these persistent implementation challenges.

Notably, interviewees linked this pattern to a lack of an early and well-defined clinical need, often because of AI and ML development occurring without sufficient input seeking from clinicians, patients, community members, and others. The lack of appropriate stakeholder involvement or the misalignment of values between stakeholders were identified as phase-1 failures that directly contribute to issues in the development and clinical implementation of AI and ML tools, including reduced clinical utility and acceptability. Interviewees agreed that research questions must be sensitive to real-world needs and contextual factors, such as the clinical environments in which health care providers and teams work, and emphasized that these considerations should remain central throughout the full course of development and implementation. These findings align with a qualitative study by Watson et al [30], in which leaders of academic medical centers described identifying a research question as an essential task that must take place before model development begins and suggested that consultation with clinicians and other stakeholders helps greatly in formulating the question [30].

### Aligning Values and Motivations: The Tension Between Innovation and Patient Care

Although modern medicine is an established field that prioritizes ethically robust advancement, AI and ML (in their current state) were described as rapidly evolving, technology-centric fields that prioritize innovation. Echoing concerns previously raised in the literature, interviewees described how these divergent priorities may lead to ethical tensions between the individuals and institutions that develop these technologies and the clinicians and patients who ultimately use them [6,21]. Interviewees perceived physicians’ motivations for using medical AI and ML as related to improving patient outcomes and lessening clinical burden, whereas the motivations among developers of medical AI and ML were viewed as more varied and not necessarily aligned with those of the end users.

Notably, a number of researchers agreed that basic, stepwise, or “boring” AI and ML research has benefits that may be undervalued in today’s research culture, in recognition of the understanding that innovation for its own sake is likely not inherently beneficial for the advancement of AI and ML in medicine. These findings reassuringly suggest that the physicians and researchers we interviewed distinguish similarly between the intellectual and moral dimensions of AI and ML research in the health care context, value cautious and measured innovation, and are generally aligned in their understanding that the chief aim of biomedical innovation is to reduce the burden of health disorders and conditions.

### Advancing Interdisciplinary Engagement: Recommendations for Strengthening Ethical Innovation in Medicine

Interviewees agreed that medical AI and ML’s success in both the short and long terms will require sustained efforts to engage a broad stakeholder base before development efforts and reimagine interdisciplinary education and training for both developers and clinicians. The value of increased and earlier stakeholder involvement has been previously identified [22,29] and was raised by many interviewees as an actionable strategy for anticipating and meeting current challenges related to problem formulation. Although AI and ML developers possess the technical expertise needed to create algorithms, clinicians possess the insight and professional experience needed to determine how best to integrate a potential tool into an existing clinical space.

As the field of medical AI and ML innovation continues to expand, participants emphasized that it should increasingly involve dual-trained individuals with expertise in both AI or ML and medicine. Expanded opportunities for the dual training of new clinician researchers are greatly needed, in addition to more interdisciplinary training for individuals whose expertise resides in a single field. This is especially relevant in light of the FDA’s 2022 guidance regarding the 21st Century Cures Act, where it was indicated that clinical decision support software is not classified as a medical device when the health care provider “can independently review the basis for [the] recommendations” [43]. Consequently, AI and ML tools that have logic and inputs that can be reviewed will likely not require the same FDA oversight as other medical devices, shifting the onus of interpreting and verifying the outputs of these tools to clinicians who may have varying levels of understanding of AI and ML technologies. Although there are still many unanswered questions regarding how the FDA’s guidance will affect hospital systems and health care providers as the availability of AI- or ML-enabled clinical tools systems increases, those who have relevant training in AI and ML will be better prepared to understand the functionality of these tools and make confident clinical decisions based on their output [44,45].

Beyond increased technical education, interviewees in this project specifically underscored the need for systematic ethics training and resources for tool developers, with both groups expressing concern regarding the lack of institutionalized ethical standards in the field of AI and ML. They suggest that the lack of ethical consensus within AI and ML may represent a limiting factor for innovation in medicine. This finding indicates that more empirical work is needed to develop a coherent and coordinated framework for reasoning through ethical problems in medical AI and ML, and to develop adequate guidelines, regulations, and safeguards that ensure medical AI and ML’s acceptability to care teams and fulfillment of public trust responsibilities. In working toward greater ethical alignment, interviewees described a myriad of questions related to phase 1 that they felt were important for medical AI and ML teams to consider before the start of algorithm design and development (Table 2).

**Table 2:** Questions to consider at the start of medical artificial intelligence (AI) or machine learning (ML) projects, as recommended by interviewees.

Need	Questions to consider	Relevant quotes
Assess motivations, priorities, and incentives	What are the motivations for the creation of this tool? Are any of these in conflict with the goals and ideals of the field of medicine? What economic or social incentives may be influencing the motivations?	“Am I doing this because I want to innovate or am I doing this because I really want to prove this one process or take care of the patient?” (participant 15, physician)
Involve stakeholders	Have the perspectives of stakeholders who may be affected by the development and use of this tool (patients, family members, clinicians, and hospital staff) been solicited and considered? Have the perspectives of diverse stakeholders been solicited and considered (individuals of different races, ethnicities, genders, sexualities, ages, SES^a^)? Have stakeholders’ concerns been addressed and their input incorporated?	“Who’s asking the questions?” (participant 14, researcher) “Who [hasn’t] had the privilege to ask the questions? Who hasn’t been empowered to be able to ask the questions?” (participant 33, ML researcher)
Identify problem space	What *specific* role will this technology fill in medicine? What is the *specific* problem in medicine that the tool will address? How is this problem currently being addressed? How may it benefit from the use of AI or ML? Has the input of stakeholders been incorporated when identifying the problem space?	“What is the problem we’re trying to solve? Think of AI as just an enabling technology like anything else...You should never do something for the sake of using AI.” (participant 15, physician)
Evaluate need	Can this problem be solved without AI or ML? Is AI or ML the best tool currently available to address this problem? What are some possible non-AI or non-ML solutions for this problem? Are these more practical, feasible, affordable, accessible? Has the input of stakeholders been incorporated when evaluating the necessity of the AI or ML solution?	“Does machine learning actually make the application or the intervention more effective? Do we need to use machine learning?...When does machine learning actually improve things, and when should you maybe not use machine learning or refuse the use of machine learning if it can actually do more harm than good?” (participant 22, physician).
Assess collaborations	Has an interdisciplinary team of collaborators been established? Does the team have the expertise in medicine needed to be able to thoughtfully develop this tool? Will these collaborations be able to continue as the project progresses? Do the collaborators include different types of stakeholders?	“[Talk] to different stakeholders to see what things they find as issues, either in the workplace or with their profession, that AI could really assist with. That collaboration...[ensures] that it is something that is actually useful in the medical and healthcare field.” (participant 27, physician)
Push boundaries on interdisciplinary knowledge	What should interdisciplinary knowledge look like? What assumptions about interdisciplinary knowledge and collaboration should be reexamined or challenged in this emerging context?	“I cannot stress enough how important it is to have more people who are dual trained: who deeply understand the subject matter and who deeply understand algorithms...Team science is great but in order to do really transformational work, you need some of both on some level.” (participant 33, ML researcher)

^a^SES: socioeconomic status.

### Limitations

This study had several limitations. Owing to their qualitative nature, the results are not representative; however, the ethical issues raised could be transferable to other similar areas of study in medicine. In addition, because the semistructured design of the interviews emphasized open-ended questions, the ability to compare responses among and between the participant groups was limited. Furthermore, this analysis did not include the perspectives of other potential stakeholder groups, such as patients, ethicists, industry researchers, representatives, or other health care professionals beyond physicians. Additional qualitative and quantitative research is required to confirm these findings. Research involving complementary quantitative approaches could be useful once ethical concerns are articulated, refined, and prioritized. Vignette studies such as surveys that present hypothetical scenarios offer a promising approach to support reproducibility. Future stakeholder studies may benefit from focusing on the “problem formulation” phase of research, as it presents an early opportunity to avoid costly failures during development and implementation.

### Conclusions

In conclusion, this study provides a description of the nuanced views of researchers and physicians regarding ethical considerations in the use of AI and ML technologies in medicine. Considerations related to the earliest processes in a medical AI or ML project, such as selecting a research question and forming a research team, were highlighted by interviewees for their potential to have an outsized impact on the following phases of development and implementation. The phase-1 ethical issues identified by interviewees were notably interdisciplinary in nature and invited questions regarding how to align priorities and values across disciplines and ensure clinical value throughout the development and implementation of medical AI and ML. Relatedly, interviewees suggested interdisciplinary solutions to these issues, for example, more resources to support knowledge generation and collaboration between developers and physicians, engagement with a broader range of stakeholders, and efforts to increase diversity in research both broadly and within individual teams. Although some of the issues addressed in this paper may be outside the control of any individual researcher or team, thorough individual- or team-level assessment of these considerations before the development phase could aid in maximizing the benefit of new tools for patients and care teams and ultimately increase the successful uptake of AI and ML innovations.

## Abbreviations

AI: artificial intelligence
FDA: Food and Drug Administration
ML: machine learning

## Data Availability

The full data set is not available to protect the identities of the participants. A limited data set is available upon request.
